# IDO1 facilitates esophageal carcinoma progression by driving the direct binding of NF-κB and CXCL10

**DOI:** 10.1038/s41420-023-01689-3

**Published:** 2023-10-31

**Authors:** Wenjian Yao, Xiaohai Cui, Haodong Peng, Yongkang Zhang, Xiangbo Jia, Sen Wu, Jian Zhao

**Affiliations:** 1 Department of Thoracic Surgery, Henan Provincial People’s Hospital, People’s Hospital of Zhengzhou University, School of Clinical Medicine, Henan University, No.7 Weiwu Road, Zhengzhou, 450003 Henan province China; 2 Department of Thoracic Surgery, The first affiliated hospital of xi’an jiaotong university, No.277 Yanta West Road, Xi’an, 710061 Shanxi province China; 3 Department of Thoracic Surgery, Zhengzhou University People’s Hospital, Henan Provincial People’s Hospital, No.7 Weiwu Road, Zhengzhou, 45003 Henan province China; 4 Department of Thoracic Surgery, Liaoning Cancer Hospital, No.44-3 Xiaohe Yan Road, Dadong District, Shenyang, 110000 Liaoning Province China

**Keywords:** Oesophageal cancer, Cell biology

## Abstract

Esophageal carcinoma (EC), one of the most lethal human malignancies, lacks effective targeted therapies. Indoleamine 2,3-dioxygenase 1 (IDO1) plays a key role in a variety of cancers, but its role and mechanism in EC are still unclear. Immunohistochemistry and qRT-PCR were used to analyze the expression of IDO1 in EC, and the prognostic value of IDO1 in EC was evaluated by Kaplan-Meier test. The in vitro and in vivo function loss/acquisition tests were performed to evaluate the biological effects of IDO1 in EC. The mechanism of action of IDO1-regulation EC was explored through Firefly luciferase & Renilla luciferase activity reporter, chromatin immunoprecipitation (ChIP) and immunofluorescence (IF) assays. Clinically, IDO1 expression was abnormally elevated in EC and positively correlated with overall survival. Functionally, IDO1 was contributed to the proliferation and migration of EC cells. Mechanically, IDO1 regulated the expression of chemokine C-X-C ligand 10 (CXCL10) by promoting the entry of NF-κB into the nucleus to combine with the promoter of CXCL10. Consistently, IDO1 facilitated EC progression may dependent on the presence of CXCL10. Moreover, NF-κB alleviated the inhibitory effect of IDO1 knockdown on EC. IDO1 drove the progression of EC by directly binding NF-κB and CXCL10, the finding that may provide an effective theoretical basis for precise therapies for EC.

## Introduction

Esophageal carcinoma (EC) represents an aggressive malignancy that is histologically divided into esophageal squamous cell carcinoma (ESCC) and esophageal adenocarcinoma (EAC) [1]. EC has clinical features of late diagnosis, metastasis, treatment resistance and frequent recurrence [2]. Currently, surgical resection, radiotherapy and chemotherapy are the main treatments for EC [3]. In advanced EC, treatment mainly consists of cytotoxic drugs [4]. The first-line treatment regimen is usually pyrimidine (5-fluorouracil or capecitabine) combined with oxaliplatin or cisplatin, and single-agent chemotherapy with Taxane or Irinotecan is a common second-line treatment option [1]. However, even with treatment according to standard treatment guidelines, the overall survival of patients with advanced EC is unsatisfactory [4]. Targeted therapy has received increasing attention in recent years with the comprehensive genomic characterization of EC [5]. Cetuximab, bevacizumab, and pembrolizumab have been shown to play an important role in the treatment of EC [6]. However, available targeted therapies for EC are still lagging behind, and the adverse events, optimal doses, and effective combinations still require further exploration.

Indoleamine 2,3-dioxygenase 1 (IDO1, Gene ID: 3620) also known as: IDO, IDO-1, INDO, is a rate-limiting metabolic enzyme that converts the essential amino acid tryptophan (Trp) to the downstream catabolite of kynurenine [7]. Interestingly, many studies have shown that IDO1 is highly expressed in various types of human cancers [8]. Multiple lines of evidence link IDO1 expression with poor prognosis in patients, including those diagnosed with acute myeloid leukemia [9], non-small cell lung cancer [10], colorectal cancer [11], prostate cancer [12], endometrial cancer [13], ovarian cancer [14], and EC [15]. Unexpectedly, higher IDO1 expression levels in patients with hepatocellular carcinoma [16] and renal cell carcinoma [17] were associated with better survival outcomes. Given the contradictory relationship between IDO1 expression and prognosis in different cancers, its biological functions in cancer have been extensively studied. Importantly, TDO1 contributes to inflammation, angiogenesis and immune escape to promote tumor cell survival, growth and metastasis [8, 18, 19]. Passarelli et al., proposed that targeting the immune metabolism mediated by the IDO1 pathway may contribute to the immune resistance of endometrial cancer [20]. Therefore, inhibitors targeting IDO1 are recognized as a promising direction for cancer immunotherapy [21, 22]. Nonetheless, the link between IDO1 and EC has not yet been elucidated and much research is needed. In this study, the progression of IDO1 regulation of EC has been explored at the clinical, cellular and molecular levels, and the results would lay a theoretical foundation for the targeted therapy of this disease.

## Results

### IDO1 is abundantly expressed in EC

**Fig. 1:**
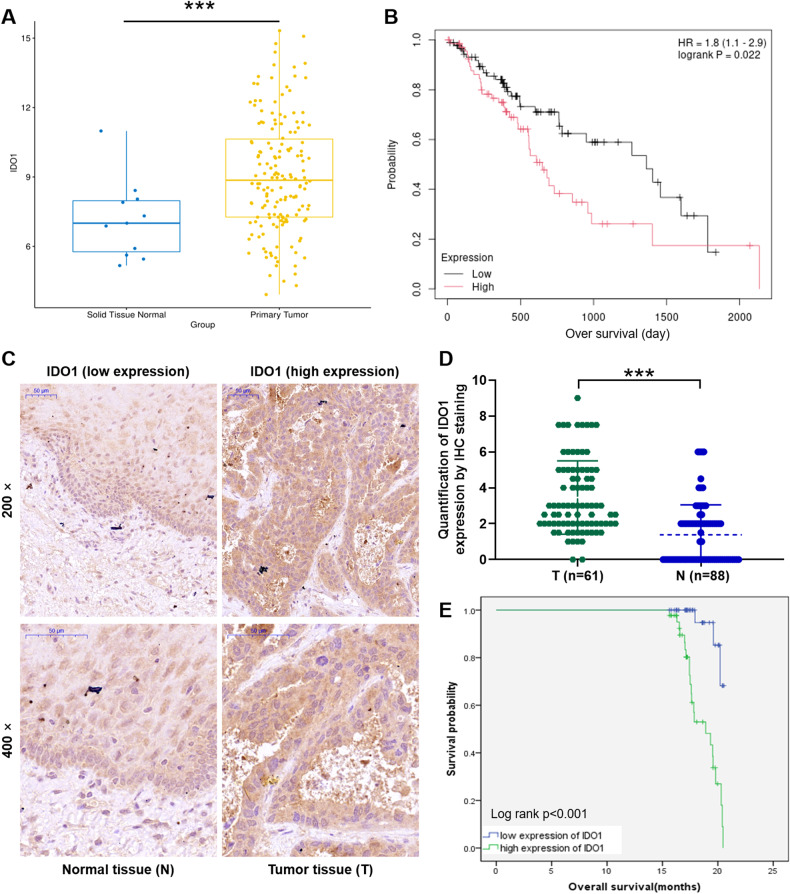
Correlation between IDO1 expression level and EC. **A** The expression of IDO1 in tumor tissue (*n* = 162) and normal tissue (*n* = 11) samples was analyzed based on The Cancer Genome Atlas (TCGA)-EC database. **B** Based on the information of TCGA database, the correlation between IDO1 expression and survival was analyzed. **C** Representative diagrams of immunohistochemical staining to reveal the expression of IDO1 in EC tissues and normal tissues. **D** Quantitative results of immunohistochemical staining of IDO1 expression in EC tumor tissue (T, *n* = 61) and normal tissue (*N*, *n* = 88). **E** A correlation analysis of high or low expression of IDO1 with overall survival in EC patients based on Kaplan-Meier method (*p* < 0.001). ***p < 0.001.

The RNAseq from tumor tissue (*n* = 162) and normal tissue (*n* = 11) samples based on The Cancer Genome Atlas (TCGA) database was analyzed and founding that IDO1 was generally highly expressed in EC (*P* < 0.001; Fig. 1A). Subsequently, prognostic analysis based on the clinical information of TCGA samples revealed that the expression level of IDO1 was significantly correlated with the overall survival of EC (*P* = 0.022; Fig. 1B). To verify IDO1 expression level was correlated with EC, we prepared tissue microarrays based on tumor tissues (*n* = 61) and normal tissues (88) of clinical patients and performed immunohistochemistry to explore the expression of this gene. As showed in Fig. 1C, the signal intensity (tan staining) of IDO1 in tumor tissues was more intense than that in normal tissues. Moreover, immunohistochemistry of all tissues showed a score higher than 3 was defined as high expression of IDO1, otherwise low expression. Our data indicated that the proportion of IDO1 high expression in tumor tissues of EC patients was significantly higher than that in normal tissues (*P* < 0.001; Fig. 1D and Table 1). The above date demonstrated the high expression of IDO1 in EC.

**Table 1:** Expression patterns in EC tissue and normal esophageal tissue was revealed by immunohistochemistry analysis.

IDO1 expression	EC tissue		Normal esophageal tissue		*p*-value
	Cases	Percentage	Cases	Percentage	
Low	28	45.9%	69	82.3%	< 0.001
High	33	54.1%	19	17.7%	

### Correlation between IDO1 expression level and EC

Furthermore, the relationship between IDO1 expression and tumor characteristics in patients with EC (*n* = 61) was analyzed by Mann-Whitney U. The data demonstrated that expression of IDO1 was positively correlated with lymph node metastasis (N-value; *P* = 0.010) (Table 2). Consistently, Spearman correlation coefficient further proved the above results (Table 3). These findings suggested that increased expression of IDO1 may be accompanied by the deepening of tumor malignancy in EC patients. Additionally, Kaplan-Meier test was used to calculate the correlation between IDO1 expression and overall survival of EC patients. Our results revealed that EC patients with high IDO1 expression had significantly shorter overall survival than those with low IDO1 expression (*P* < 0.001; Fig. 1E). Taken together, IDO1 was highly expressed in EC and was associated with malignant progression of the disease, which may serve as a clinical prognostic marker.

**Table 2:** Relationship between IDO1 expression and tumor characteristics in patients with esophagus cancer.

Features	No. of patients	IDO1 expression		*p*-value
		low	high	
All patients	61	28	33	
Age (years)				0.907
≤ 65	30	14	16	
> 65	31	14	17	
Gender				0.221
Male	48	24	24	
Female	13	4	9	
T Infiltrate				0.208
T0	1	1	0	
T1	2	0	2	
T2	13	8	5	
T3	32	15	17	
T4	13	4	9	
lymphatic metastasis (*N*)				0.010
N0	26	16	10	
N1	15	7	8	
N2	11	4	7	
N3	9	1	8	
Stage				0.084
I	2	1	1	
II	26	16	10	
III	30	9	21	
IV	3	2	1	
Metastasis				0.122
M0	59	26	33	
M1	2	2	0

**Table 3:** Relationship between IDO1 expression and tumor characteristics in patients with esophagus cancer.

		IDO1
lymphatic metastasis (*N*)	Spearman correlation coefficient	0.333
	Significance (double tail)	0.009
	*N*	61

### IDO1 contributes to the proliferation and migration of TE-1 and ECA-109

**Fig. 2:**
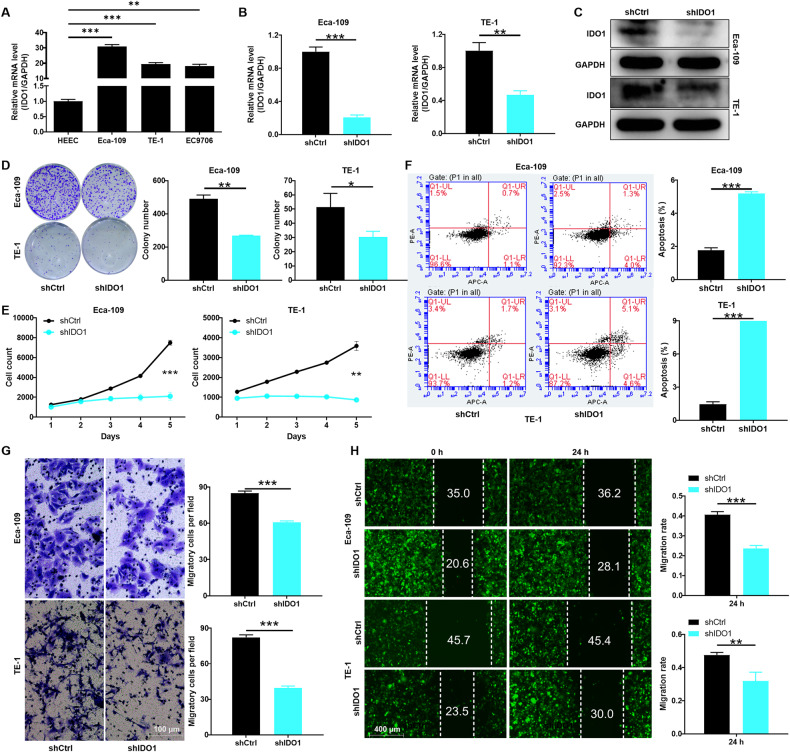
Effect of reduced IDO1 expression on EC cell phenotypes. **A** The relative mRNA expression of IDO1 in human EC cell lines and normal esophageal epithelial cells HEEC was evaluated by qRT-PCR. **B** The silencing efficiency of IDO1 was detected by qRT-PCR after the small hairpin RNA (shRNA) interferes with the expression of IDO1 in TE-1 and ECA-109 cells. **C** After shRNA interfered with IDO1 expression in EC cells, its protein expression was detected by western blotting. **D**, **E** Effect of IDO1 expression reduction on (**D**) clone formation and (**E**) proliferation in TE-1 and ECA-109 cells. **F** The effect of decreased expression of IDO1 in EC cells on their apoptosis was detected by flow cytometry. **G**, **H** EC cells with reduced IDO1 expression underwent (**G**) Transwell experiments and (**H**) wound healing experiments to assess migration, respectively. The data are represented as the mean ± SD. *p < 0.05, **p < 0.01, ***p < 0.001.

To further clarify the role of IDO1 in EC, we performed a series of functional assays at the cellular level. As expected, the relative mRNA expression of IDO1 in EC cell lines TE-1 and ECA-109 was significantly higher than that in human normal esophageal epithelial cells HEEC (*P* < 0.001; Fig. 2A). Subsequently, the expression of IDO1 in TE-1 and ECA-109 cells was disrupted by lentiviral mediation. Not surprisingly, the mRNA expression of IDO1 in lentiviral shIDO1 transduced TE-1 and ECA-109 cells was significantly lower than that of negative control shCtrl cells (*P* < 0.01; Fig. 2B). Consistently, the protein expression of IDO1 in TE-1 and ECA-109 cells of shIDO1 group was downregulated compared with shCtrl cells (Fig. 2C).

Next, the loss-of-function assays were conducted in lentiviral shIDO1 or shCtrl transduced TE-1 and ECA-109 cells to determine the effect of IDO1 on EC. Compared to negative control shCtrl cells, TE-1 and ECA-109 cells in the shIDO1 group produced smaller and fewer cell clones (*P* < 0.05; Fig. 2D). After 5 days of counting EC cells, the proliferation ability of TE-1 and ECA-109 cells in shIDO1 group was sharply decreased compared with that in shCtrl group (*P* < 0.01; Fig. 2E). Moreover, to investigate the effect of abnormal ID01 expression on apoptosis of EC cells, we performed corresponding detection based on flow cytometry. The data indicated that the apoptosis rate of TE-1 and ECA-109 cells was significantly enhanced when ID01 expression was decreased (*P* < 0.001; Fig. 2F). Moreover, Transwell assay showed that the migration ability of TE-1 and ECA-109 cells was markedly inhibited when ID01 expression was abnormally decreased (*P* < 0.001; Fig. 2G). As expected, the wound healing experiment confirmed the above phenomenon again (*P* < 0.01; Fig. 2H). Collectively, the above results suggested that IDO1 may functioned as an oncogene, which was crucial for the proliferation and migration of EC cells.

### IDO1 drives direct binding of NF-κB and CXCL10 in EC cells

**Fig. 3:**
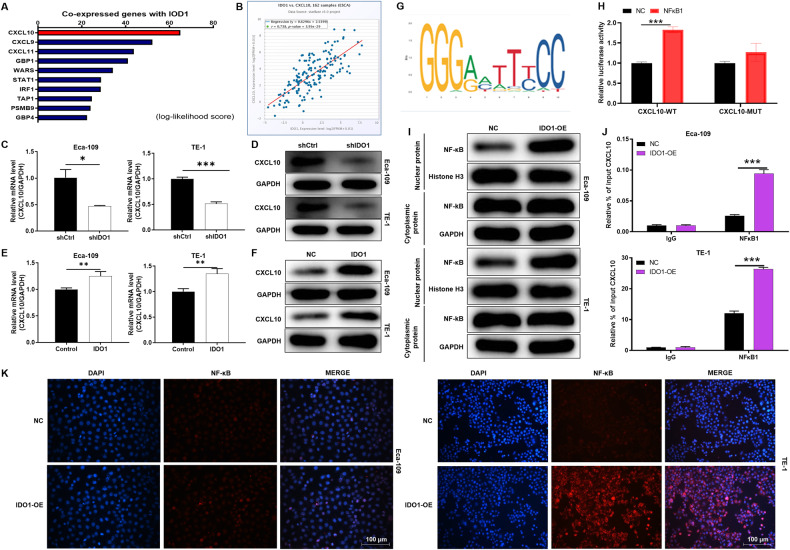
IDO1 drives direct binding of NF-κB and CXCL10 in EC cells. **A** IDO1 was co-expressed with CXCL10 based on bioinformatics analysis. **B** There was a significant positive correlation between IDO1 and CXCL10 expression. **C**, **D** Effect of IDO1 knockdown on mRNA and protein expression of CXCL10 in cell lines TE-1 and ECA-109. **E**, **F** IDO1 overexpression level can upregulate the expression of CXCL10 in EC cells. **G** According to the JASPAR CORE database, we predicted and found that CXCL10 existed binding sequence sites with transcription factor NF-κB1. **H** The wild type (CXCL10-WT) and mutant (CXCL10-MUT) of transcription factor binding motif were produced and transfected with NF-κB1 overexpression (NF-κB1) and negative control (NC) cells to evaluated Firefly luciferase & Renilla luciferase activity. **I** The effect of IDO1 overexpression on the expression of NF-κB protein in the nucleus and cytoplasm of esophageal carcinoma cells was investigate by western blotting. **J** We performed ChIP assay and verified that IDO1 overexpression can promote the binding of transcription factor NF-κB1 to the promoter of CXCL10. **K** IF assays demonstrated that the overexpression of IDO1 could drive NF-κB to enter the nucleus relative to the control. The data are represented as the mean ± SD. *p < 0.05, ***p < 0.001.

Based on bioinformatics analysis (https://www.coexpedia.org/search.php), we found that IDO1 was co-expressed with CXCL10 (Fig. 3A). Further analysis revealed a significant positive correlation between IDO1 and CXCL10 expression levels (*P* < 0.001; Fig. 3B). In addition, IDO1 knockdown could down-regulate CXCL10 mRNA and protein expression in EC cells (*P* < 0.05; Fig. 3C, D). Consistently, IDO1 overexpression level can upregulate the expression of CXCL10 (*P* < 0.05; Fig. 3E, F). Based on the above results, we speculated that there might be some special molecular regulatory mechanism between IDO1 and CXCL10.

According to the JASPAR CORE database, we predicted and found that CXCL10 existed binding sequence sites with transcription factor NF-κB1 (Fig. 3G). Subsequently, the wild type (CXCL10-WT) and mutant (CXCL10-MUT) of transcription factor binding motif were produced and transfected with NF-κB1 overexpression (NF-κB1) and negative control (NC) cells, respectively. By Firefly luciferase & Renilla luciferase activity reporter assay, NF-κB1 overexpression could significantly up-regulate the expression of luciferase of CXCL10-WT rather than CXCL10-MUT, suggesting that NF-κB1 and CXCL10 possessed a direct binding effect (*P* < 0.001; Fig. 3H). Subsequently, the effect of IDO1 overexpression on the expression of NF-κB protein in the nucleus and cytoplasm of EC was investigated. These results suggested that IDO1 overexpression promoted NF-κB transfer from cytoplasm to nucleus (Fig. 3I). Additionally, we performed ChIP assay in Eca-109 and TE-1 and verified that IDO1 overexpression could promote the NF-κB to the CXCL10 promoter (*P* < 0.001; Fig. 3J). Moreover, the effect of NF-κB overexpression on IDO1-induced CXCL10 expression was detected by western blotting experiments. The results showed that NF-κB could up-regulate the expression of CXCL10 (Fig. S1). This analysis will provide evidence of NF-κB-mediated transcriptional regulation of CXCL10 expression. Interestingly, IF assays demonstrated that the overexpression of IDO1 could drive NF-κB to enter the nucleus relative to the control (Fig. 3K). As a consequence, these results revealed that IDO1 regulated the expression of CXCL10 by promoting the entry of NF-κB into the nucleus to combine with the promoter of CXCL10.

### IDO1 promotes EC progression dependent on the presence of CXCL10

**Fig. 4:**
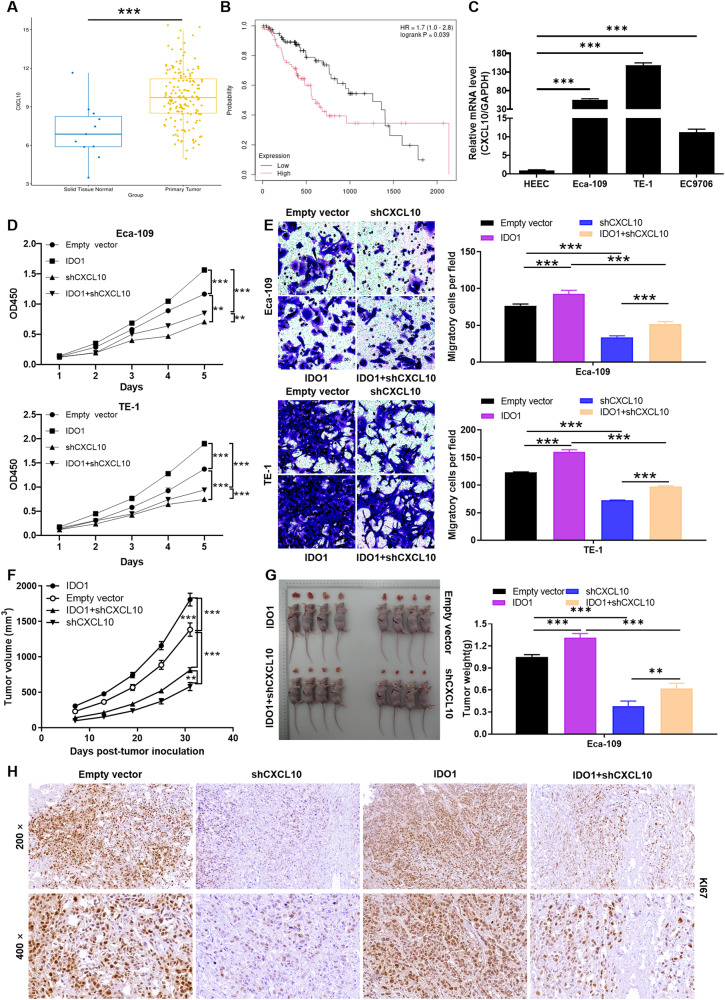
IDO1 promotes EC progression dependent on the presence of CXCL10. **A**, **B** According to the information of TCGA database, the expression of IDO1 in EC and its correlation with survival were analyzed. **C** The relative mRNA expression of CXCL10 in human EC cell lines as well as normal esophageal epithelial cells was evaluated by qRT-PCR. **D**, **G** The synergy between IDO1 and CXCL10 in EC cell models was assessed through (**D**) cell counting, (**E**) Transwell, and (**F**, **G**) mice xenograft experiments, respectively. **H** Representative diagrams of immunohistochemical staining to reveal the expression of KI67 in mice tumor tissues. Empty vector was used as a negative control, IDO1 was overexpressing IDO1 in EC cells, shCXCL10 was down-regulating CXCL10 expression, IDO1+shCXCL10 was simultaneously down-regulating CXCL10 and overexpressing IDO1. The data are represented as the mean ± SD. **p < 0.01, ***p < 0.001.

According to TCGA database, we found that CXCL10 expression was generally elevated in tumor tissue (*n* = 162) than that in normal tissue (*n* = 11) samples (*P* < 0.001; Fig. 4A). Subsequently, the prognostic analysis based on the clinical information of TCGA indicated that the expression level of IDO1 was significantly correlated with the overall survival of EC (*P* = 0.038; Fig. 4B). Moreover, the relative mRNA expression of CXCL10 in EC cell lines, especially in TE-1 and ECA-109 was significantly higher than that in human normal esophageal epithelial cells HEEC (*P* < 0.001; Fig. 4C). To further elucidate the effects of IDO1 and CXCL10 on the regulation of EC, we performed cell functional recovery experiments in vitro and in vivo. Here, empty vector was used as a negative control, IDO1 was overexpressing IDO1 in EC cells, shCXCL10 was down-regulating CXCL10 expression, IDO1+shCXCL10 was simultaneously down-regulating CXCL10 and overexpressing IDO1. As showed in Fig. 4D, the proliferation ability of IDO1 overexpressed cells was significantly enhanced compared with that of control cells (*P* < 0.001). Down-regulation of CXCL10 significantly attenuated cell proliferation compared with the control (*P* < 0.001). Interestingly, down-regulation of CXCL10 attenuated the promotion of proliferation by IDO1 overexpression (*P* < 0.001). Moreover, the migration ability of EC cells was enhanced when IDO1 was overexpressed, and this effect was alleviated by down-regulation of CXCL10 (*P* < 0.001; Fig. 4E). Additionally, the regulation of IDO1 and CXCL10 expression abnormalities on the tumorigenesis ability of EC cells was verified in xenograft models. Measurement of tumor volume and body weight revealed that IDO1 overexpression drove tumor growth, whereas CXCL10 knockdown showed an inhibitory effect (*P* < 0.01; Fig. 4F, G). Consistently, tumor tissues in IDO1 overexpression and CXCL10 knockdown groups showed high and low expression of KI67, respectively (Fig. 4H). Therefore, IDO1 promoted EC progression may dependent on the presence of CXCL10.

### NF-κB alleviated the inhibitory effect of IDO1 knockdown on EC

**Fig. 5:**
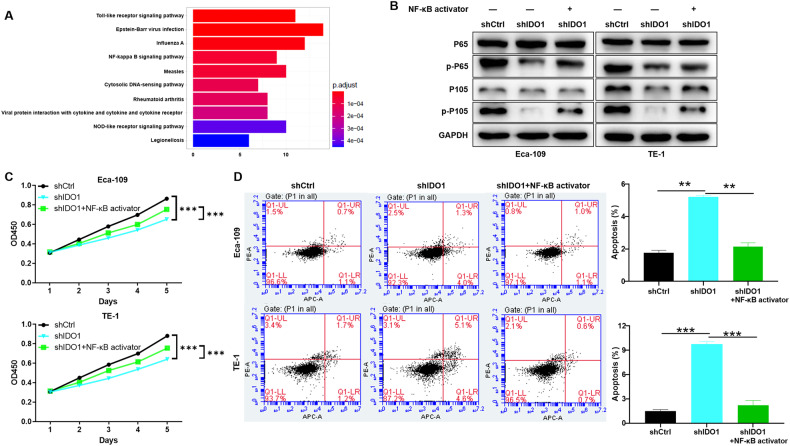
NF-κB alleviated the inhibitory effect of IDO1 knockdown on EC. **A** The classical pathway enrichment KEGG analysis was performed on genes co-expressed with IDO1 and found that NF-κB signaling pathway was significantly enriched. **B** Western blotting was performed in EC cells (shCtrl vs shIDO1) to detect protein expression of typical components of NF-κB signaling pathway. **C**, **D** EC cells with IDO1 knockdown were treated with NF-κB activator and subjected to proliferation and apoptosis detection, respectively. The data are represented as the mean ± SD. *p < 0.05, **p < 0.01, ***p < 0.001.

Here, we performed classical pathway enrichment KEGG analysis on genes co-expressed with IDO1 and found that NF-κB signaling pathway was significantly enriched (Fig. 5A). To validate our analysis results, western blotting was performed in EC cells (shCtrl vs shIDO1) to detect protein expression of typical components of NF-κB signaling pathway. The data indicated that IDO1 knockdown attenuated the phosphorylation of p65 and p105, which was alleviated by NF-κB activator treatment (Fig. 5B). These results suggested that NF-κB may be involved in IDO1 regulation of EC. Furthermore, EC cells with IDO1 knockdown were treated with NF-κB activator and subjected to proliferation and apoptosis detection, respectively. As shown in the Fig. 5C, D, treatment with NF-κB activator partially reversed the regulation of proliferation inhibition (*P* < 0.001) and apoptotic promotion (*P* < 0.05) in EC cells induced by IDO1 knockdown. As a result, IDO1 promoted the progress of EC may via NF- κB signaling pathway.

## Discussion

EC, one of the most lethal human malignancies, lacks effective targeted therapies [4]. Notably, multiple lines of evidence link IDO1 expression with poor prognosis in patients with cancers [8–17]. Recently, Lynch et al., revealed that decreased IDO1 expression in melanoma metastases was associated with improved overall survival [23]. Additionally, Wei et al. reported that IDO1 up-regulation induced immunosuppression in triple-negative breast cancer [24]. Given the relationship between IDO1 expression and various types of human cancers, its biological functions and mechanism in EC were investigated. Our data demonstrated that IDO1 expression was abnormally elevated in EC and positively correlated with overall survival. In addition, the progression of IDO1 regulation of EC had been explored at the cellular and molecular levels. The results indicated that IDO1 was contributed to the proliferation and migration of tumor cells, which may function as an oncogene in EC.

Furthermore, we found that IDO1 was co-expressed with chemokine C-X-C ligand 10 (CXCL10) based on bioinformatics analysis. CXCL10, also known as interferon-γ-inducible protein 10 (IP-10), is a pro-inflammatory cytokine secreted by immune cells [25]. It had been reported that CXCL10 was involved in tumor growth and metastasis, which may serve as a novel target for cancer treatment [26]. Reschke et al. indicated that CXCL10 produced by human melanoma during metastasis was a positive prognostic factor for the response to immunotherapy for the disease [27]. Wu et al. proposed that CXCL10 promoted the proliferation of Tamoxifen-resistant breast cancer cells through the AKT pathway [28]. Wang et al. indicated that tumor necrosis factor-α (TNF-α) activates PI3K/AKT and p38 MAPK parallel signal transduction pathways and stimulates the downstream NF-κB pathway p65 to enter the nucleus and activate CXCL10 transcription in colon cancer cells [29]. In this study, we revealed a significant positive correlation between IDO1 and CXCL10 expression levels in EC. Mechanically, IDO1 regulated the expression of CXCL10 by promoting the entry of NF-κB into the nucleus to combine with the promoter of CXCL10. Functionally, IDO1-facilitated EC progression may dependent on the presence of CXCL10.

Moreover, we performed classical pathway enrichment KEGG analysis on genes co-expressed with IDO1 and found that NF-κB signaling pathway was significantly enriched. The nuclear factor NF-κB pathway had long been recognized as a typical proinflammatory signaling pathway [30]. For example, the activation of NF-κB signaling promoted the invasion and metastasis of lung cancer [31]. In addition, DHX9 induced the malignant phenotype of colorectal cancer by activating NF-κB signaling pathway [32]. Furthermore, NF-κB signaling pathway was involved in focal adhesion kinase activation to promote the invasion and metastasis of non-small cell lung cancer [33]. Here, we identified that NF-κB could alleviate the inhibitory effect of IDO1 knockdown on EC. As a result, IDO1 promoted the progress of EC may via NF-κB signaling pathway.

Clinically, IDO1 expression was abnormally elevated in EC and positively correlated with overall survival. Functionally, IDO1 was contributed to the proliferation and migration of EC cells. Mechanically, IDO1 regulated the expression of CXCL10 by promoting the entry of NF-κB into the nucleus to combine with the promoter of CXCL10. In summary, IDO1 drove the progression of EC by directly binding NF-κB and CXCL10, the finding that may provide an effective theoretical basis for precise therapies for EC.

## Materials and methods

### Samples collection and ethical statement

The tumor tissues and normal tissues were obtained from patients with EC who underwent surgical resection, which were purchased from Shanghai Outdo Biotech Company. The inclusion criteria of EC patients were that they not receive radiotherapy or chemotherapy before operation. The specimens were used with written informed consent from the patients and approval by the Ethics Committees of the Henan Provincial People’s Hospital, Zheng Zhou University (NO. 2021202).

### Immunohistochemistry

Tissue microarrays consisting of EC tissue (*n* = 61) and normal esophageal tissue (*n* = 88) were fabricated according to the procedures provided in the literature [34]. Briefly, tissue slides were dewaxed with xylene, washed with alcohol, repaired with citrate, and blocked with hydrogen peroxide and 5% serum. The tissues were incubated with primary antibody (anti-IDO1, 1:200, Abcam, Cambridge, UK) at 4 °C overnight, then cleaned with 1×PBST buffer and incubated with secondary antibody (goat anti-rabbit IgG, 1:400, Beyotime, Shanghai, China) for 1 h at room temperature. Tissue slides were stained with DAB and hematoxylin to avoid light and then sealed with neutral gum. Finally, two pathologists scored independently according to the rate of positive cells and the intensity of staining color. Scores above the median were defined as high expression, otherwise low expression.

### Cell culture

Human normal esophageal epithelial cells HEEC, EC cell lines TE-1, ECA-109 and EC9706 (American Type Culture Collection; Manassas, VA, USA) cells were kept at 37 °C with 5% CO_2_ and cultured in Dulbecco’s modified Eagle’s medium (DMEM; HyClone, Logan, UT, USA) supplemented with 10% fetal bovine serum (FBS; Grand Island, NY, USA). All cells were tested for STR profiling and mycoplasma contamination.

### Cell transduction

To silence the expression of IDO1 in the EC cell lines, the small hairpin RNA (shRNA) sequence (shIDO1: 5′-TCGAGAAAGAGTTGAGAAGTT-3′) and Scramble sequence (negative control, shCtrl: (5′-TTCTCCGAACGTGTCACGT-3′) were synthesized. Next, these sequences were inserted separately into lentiviral vector BR-V108 with a green fluorescent tag (YBR, Shanghai, China). Afterwards, recombinant lentiviral plasmids were transduced into ECA-109 and TE-1 cells with Lipofectamine^®^ 3000 (Thermo Fisher Scientific, NY, USA) according to the manufacturer’s instructions. As described previously, overexpression of IDO1 (IDO1: 5′-TCGAGAAAGAGTTGAGAAGTT-3′) and knockdown of CXCL10 (shCXCL10: 5′-GAAATTATTCCTGCAAGCCAA-3′) were constructed in EC cell lines, respectively. The success of cell transduction was judged according to the fluorescence rate, knockdown efficiency was detected by qRT-PCR and western blotting.

### Quantitative reverse transcription-polymerase chain reaction (qRT-PCR)

The cellular plasmid was obtained according to the instructions provided with the Plasmid extraction kit (Takara Bio, Kyodo, Japan). The cDNA was obtained by reverse transcription using Promega M-MLV kit (Madison, Wisconsin, USA). According to manufacturer’s protocols, qRT-PCR was performed using SYBR green PCR master mix (Vazyme. Nanjing, China). The mRNA expression was normalized to levels of Glyceraldehyde 3-phosphate dehydrogenase (GAPDH) in each sample and calculated by the 2^–ΔΔCt^ methods. The primers as follows: IDO1: F 5′-AAGAACGGGACACTTTGC-3′ and R 5′-CATGATCGTGGATTTGGT-3′; CXCL10: F 5′-ACCAGAGGGGAGCAAAATCG-3′ and R 5′-CCATGTAGGGAAGTGATGGGAG-3′; GAPDH: F 5′-TGACTTCAACAGCGACACCCA-3′ and R 5′-CACCCTGTTGCTGTAGCCAAA-3′.

### Western blotting

EC cells protein were blotted as described in the literature [35]. In brief, protein was separated by electrophoresis on SDS-polyacrylamide electrophoresis (PAGE) gels, transferred to polyvinylidene difluoride (PVDF) membranes and blocked with blocking solution at room temperature for 1 h. The PVDF membranes were incubated with primary antibody (anti-IDO1, 1:750, Abcam, Cambridge, UK; anti-CXCL10, 1:1000, Saier, Tianjin, China; anti-P65, 1:2000, Proteintech, Wuhan, China; anti-p-P65, 1:1000, Abcam; P105, 1:1000, Abcam; anti-p-P105, 1:1000, Abcam; anti-GAPDH, 1:3000, Bioworld) at 4 °C overnight, then cleaned with 1×PBST buffer and incubated with horseradish peroxidase (HRP)-conjugated secondary antibody (goat anti-rabbit IgG/Goat Anti-Mouse, 1:3000, Beyotime, Shanghai, China) for 1 h at room temperature. After the membranes were washed in PBST, the blots were visualized by enhanced chemiluminescence ECL kit (Thermo Fisher Scientific).

### Colony-forming assay

EC cells were inoculated in triplicate in 6-well plates at a density of 500 cells per well and incubated for 2 weeks until colony formation. Cells were fixed in 1 mL 4% paraformaldehyde at room temperature for 30 min, stained with 0.5% crystal violet for 15 min, and washed with ddH_2_O. Cells were dried, photographed with a digital camera, and clones were counted.

### Celigo cell counting assay

EC cells were seeded in triplicate in 96-well plates at a density of 2000 cells per well. From the second day after plating, cells were examined by the Celigo apparatus (Nexcelom) once a day for 5 consecutive days. By adjusting the number of cells with green fluorescence in each scanning well plate, the data were counted, and the cell proliferation curve was drawn.

### Cell apoptosis analysis

EC cells were inoculated in triplicate in 6-well plates at a density of 2 ml per well and incubated for a week. According to the instructions of the apoptosis kit (eBioscience) performed the following operations. Briefly, cells were centrifuged for 5 min, the supernatant was discarded, and then washed with D-Hanks (pH = 7.2 ~ 7.4) precooled at 4 °C. The cell precipitates were washed with 1×binding buffer, centrifuged, resuspended with 200 μL 1×binding buffer, and stained with 10 μL Annexin V-APC for 15 min at room temperature avoid light. Flow cytometric analysis was performed using BD FACS Calibur (Millipore) and analyzed the data using FlowJo software (RRID:SCR_008520).

### Transwell migration assay

EC cells were seeded in 24-well plates at a density of 50,000 cells per well in the upper chamber and added 100 μl serum-free medium. After the cells were starved for 24 h, 600 μl of medium containing 30%FBS was added to the lower chamber. After 4 h, the medium in the upper chamber was removed and 400 μL of crystal violet was added to the 24-well plate. Migrating cells attached to the bottom of polycarbonate membranes were stained, photographed, and counted.

### Wound-healing assay

EC cells were inoculated in triplicate in 96-well plates at a density of 50,000 cells per well. After the cells were starved with serum-free medium, the lower central part of the 96-well plate was aligned using a scratch meter, and the scratch was formed by nudging upward and photographed (at this point recorded as 0 h). After 24 h, the 96-well plates were scanned by Cellomics (Thermo Fisher Scientific) and the migration ability of cells was analyzed according to the migration distance.

### Firefly luciferase & Renilla luciferase activity reporter assay

The wild-type and mutant sequences of NFκB binding site to the CXCL10 promoter (CXCL10-WT and CXCL10-MUT) were constructed and transfected into EC cells. Subsequently, according to the instructions of the Promega Dual-Luciferase system (Madison, Wisconsin, USA) to detected the expression of luciferase.

### Chromatin immunoprecipitation (ChIP) assay

According to the manufacturer’s instructions of Pierce Agarose ChIP Kit (Thermo Fisher Scientific) carried out ChIP assays. EC cells were fixed with formaldehyde at room temperature for 10 min, collected and digested by MNase lysis. The clipped chromatin was immunoprecipitated with anti-NFκB antibody (1:100, Proteintech, Wuhan, China), Histone H3 (D2B12) XP^®^ Rabbit mAb (2 μg, Cell Signaling Technology, Danvers, Massachusetts, USA) or Normal Rabbit IgG (2 μg, Cell Signaling Technology) overnight at 4 °C. The isolated complexes were collected with protein A/G agarose beads and eluted by culture in high salt concentration buffer at 65 °C. The enrichment of NFκB binders in the promoter region was identified by qRT-PCR. The primers as follows: CXCL10: F 5′-ACCAGAGGGGAGCAAAATCG-3′ and R 5′-CCATGTAGGGAAGTGATGGGAG-3′.

### Immunofluorescence (IF)

IF was performed according to the methods provided in the literature [36]. The antibodies and nuclear dye used in this experiment were shown below: anti-NF-κB, 1:100, Abcam; goat anti-rabbit IgG/Goat Anti-Mouse, 1:200, Beyotime, Shanghai, China; Fluoroshield sealer with DAPI, Abcam.

### Mice xenograft

All procedures involving mice and experimental protocols were approved by the Institutional Animal Care and Use Committees of the Henan Provincial People’s Hospital, Zheng Zhou University and accordance with the guide for the care and use of laboratory animals. Lentiviral transduced Eca-109 cells were subcutaneously injected into 8-week-old BALB/C nude mice (*n* = 16, Jicukang Biotechnology, Jiangsu, China) and divided into the following four groups (Empty vector, shCXCL10, IDO1, IDO1+shCXCL10). After 6 days, the weight and tumor size of the mice were measured every other week. According to the formula, the tumor volume was calculated as π/6 ×L×W×W, Where L stands for long diameter and W stands for short diameter. On day 31, the mice were sacrificed by cervical dislocation and the tumors were removed, weighed and photographed. The tumor tissues were prepared as tissue sections and subjected to immunohistochemistry to evaluate the expression of KI67 (anti-KI67, 1:100, Abcam; goat anti-rabbit IgG, 1:400, Beyotime), a cell proliferation marker.

### Statistical analysis of data

Student’s t-test (unpaired, two-tailed) was used to compare the mean value of two groups. Mann-Whitney U and Spearman correlation coefficient was used to evaluate the relationship between IDO1 expression and pathological features of EC patients. The significant correlation analysis in overall survival was calculated using the Kaplan-Meier test. Data was analyzed using the SPSS 21.0 (Chicago, IL, USA), bars and errors represent the mean ± standard deviation (SD) from three independent replicates. *P* < 0.05 was considered significant difference.

### Supplementary information

Figure S1

## Data Availability

Data will be made available on request.
