# Supplementary material for: IDO1 facilitates esophageal carcinoma progression by driving the direct binding of NF-κB and CXCL10
Source: Cell Death Discov. 2023 Oct 31;9:403. doi: 10.1038/s41420-023-01689-3 (PMC10616276; doi:10.1038/s41420-023-01689-3)
Supplement: Supplementary file 1 — Figure S1 [file 41420_2023_1689_MOESM1_ESM.docx]

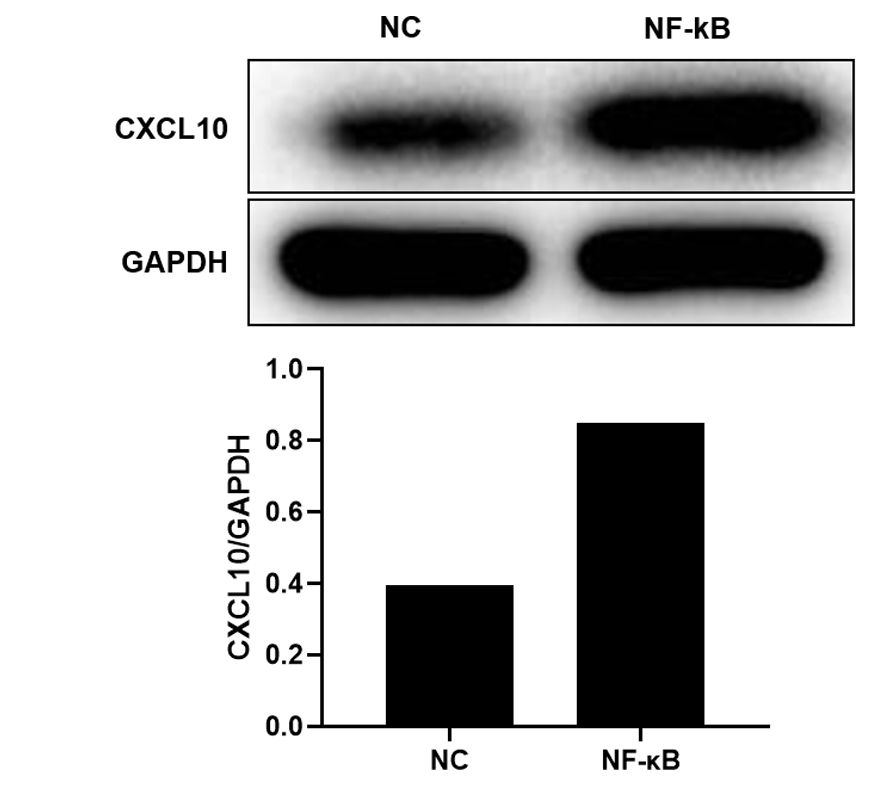


**Figure S1.** **The effect of NF-κB overexpression on IDO1-induced CXCL10 expression was detected by western blotting experiments.**
